# Terrell Medical Society

**Published:** 1896-02

**Authors:** James Orr

**Affiliations:** Secretary


### Society Notes.

### Terrell Medical Society.

Terrell Medical Society met in regular session Monday, January 6, 1896, with fifteen members in attendance, and Drs. Letcher and Church, of Dallas, and Bryant, of Plano, as visitors. Dr. W. H. Monday presiding.

The first matter of interest was the discussion of the regular question, Gonorrhœa, opened by Dr. White in an exhaustive paper covering the recent literature on the subject. General discussion was participated in by Drs. Garrett, Anthony, Stovall, Rosser, Anderson, Orr, Bass and Letcher, from which the following deductions may be made as the opinion of the Society:

That gonorrhœa has no specific treatment, is more difficult to handle than syphilis, and leaves injuries behind it from which its victims seldom or never recover.

That the disease runs a protracted course, and the cases reported as cured in a few days, are now specific urethretis.

That many cases of disease of female pelvic viscera are the sequence of gonorrhœa often contracted from husbands who supposed themselves long since well.

That many of our difficulties in the treatment of this disease grow out of the failure of the patient to appreciate the gravity of the affection, and that every physician should impress upon his patrons the magnitude of the danger threatening any one with gonorrhœa, as it is more dangerous than the bad cold with which it is often contemptuously compared.

Reports of cases being next in order, two patients were brought before the Society, one being of great interest—a case of partial paresis—affecting the whole body, as a sequelæ of typhoid fever, in a young man 22 years of age. Drs. White, Church, Bass and Rosser were of the opinion that it was a case of myelitis; that the inflammatory products were in the cord, and the prognosis, so far as it related to complete recovery, bad, but the improvement going on would probably continue till his symptoms were much better. Treatment, bichloride mercury and iodide potash, in full doses. Dr. Letcher thought the case one of loco motor ataxia, requiring powerful tonics, but with a very bad prognosis.

The other case was one of an exostosis, near the carpal end of the radius, immediate excision being recommended.

Dr. Neeley reported an interesting case of malarial hematuria, and death, which was followed by a general discussion of this disease, and the report of cases and treatment by Drs. Mathews, Garrett, Stovall, Stewart, Anthony, Rosser, Letcher and Monday. Saline purgative, with diaphoresis, was the treatment recommended, all the speakers deprecating the use of mercury and quinine.

Chronic Metritis and Endometritis was selected as the subject for discussion at the next meeting, and Dr. W. P. Dumas appointed to lead; after which an adjournment to the Harris House was taken where, in company with a number of invited guests, the annual banquet took place, which was by far one of the most enjoyable occasions in the history of the Society. Dr. F. S. White, as master of ceremonies, offered the following toasts: "Terrell Medical Society," responded to by the President, Dr. W. H. Monday; "The Medical Profession," responded to by Dr. James Orr; "The Press," responded to by Mr. Robert Carver, editor of the Terrell *Register;* "The New Woman, By A Woman," responded to by Mrs. H. C. Ryan; "Our Guests," responded to by Hon. W. H. Allen. Letters of regret from Dr. P. C. Coleman, President State Association; Dr. and Mrs. B. F. Eads, of Marshall, and Dr. L. E. Griffith, of this city, were read.

After more than two hours being spent at the table, final adjournment took place at 5 p. m.; and Terrell Medical Society starts on the sixteenth year of its existence with an earnest, faithful and united membership, believing in the efficacy of organization, and devoted to the science of medicine and the higher education of its votaries.

JAMES ORR, M. D., Secretary.

